# Personal and ambient PM_2.5_ exposure assessment in the city of Agra

**DOI:** 10.1016/j.dib.2015.12.040

**Published:** 2016-01-06

**Authors:** M. Habil, D.D. Massey, A. Taneja

**Affiliations:** a Department of Chemistry, St. John׳s College, Agra 282002, U.P, India; b Department of Chemistry, Dr. B. R. Ambedkar University, Agra 282002, India

**Keywords:** Personal exposure, PM_2.5_, Ambient concentration, Correlation analysis, Health effects

## Abstract

Human exposure to fine particles can have significant harmful effects on the respiratory and cardiovascular system. To investigate daily exposure characteristics to PM_2.5_ with ambient concentrations in an urban environment, a personal exposure measurements were conducted for school children, office workers and at their residents, in the city of Taj ‘Agra’, India. In order to account for all the sources of particulate matter exposure, measurements on several different days during December 2013 to February 2014 were carried out. Personal environment monitors (PEM) and APM 550 were used to measure PM_2.5_ concentration. The research findings provide insight into possible sources and their interaction with human activities in modifying the human exposure levels.

**Specification table**

**Table:** 

Subject area	Environmental chemistry
More specific subject area	PM_2.5_ sampling for personal and ambient concentrations in homes, schools and offices and their sources characterisation
Type of data	Text file and figures.
How data were acquired	Values were acquired used through the samplers (Personal Environmental Monitor, SKC Inc., USA) for personal sampling and for ambient (APM 550, Envirotech). Analysis for trace metal was done on Atomic Absorption Spectrophotometer (AAS) (Perkin Elmer, AAnalyst 100).
Data format	Analyzed
Data source location	The samples were collected from two homes, two schools and two offices of Agra city.
Data accessibility	The data are within this paper

**Value of data**

• To provide an in depth understanding on personal monitoring of fine particle concentrations affected by different indoor and outdoor environments.
• To provide methodological approaches to assess population exposures and risk assessment related to particulate pollutant.
• The values provided gives an initial estimates of emissions and their implications, which can be a useful addition to the existing literature, in particular for a country like India; where such measurements are yet under-represented.

**Data**

Personal and ambient monitoring for PM_2.5_ was carried in the homes, schools and offices of the city Agra city to know their possible sources and their effects upon the occupants.

## 1. Experimental design, materials and methods

### 1.1. Sample collection

**Fig. 1:**
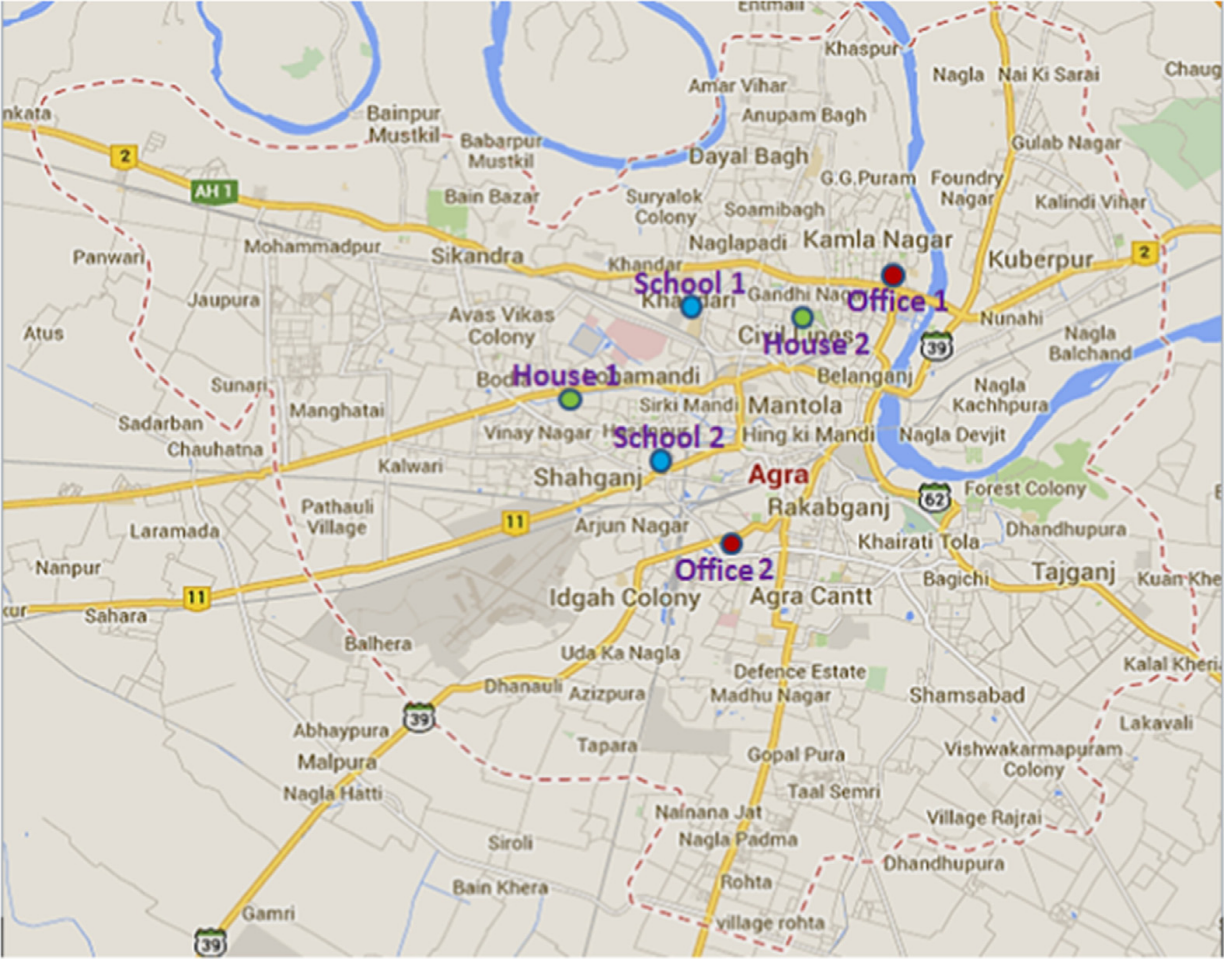
Map of Agra showing sampling sites.

**Fig. 2:**
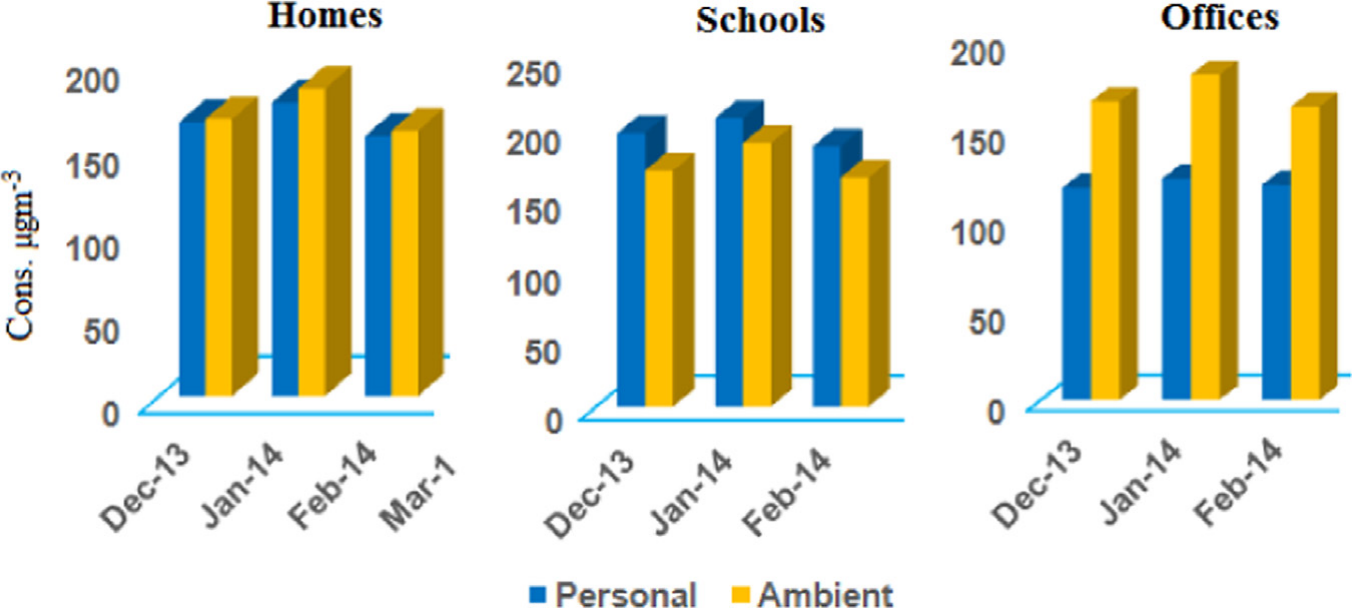
Month Concentration of PM_2.5_ in μg m^−3^ for personal and ambient monitoring at homes from December 2013 to February 2014.

The values for mass and metal concentrations of PM_2.5_ were monitored in personal and ambient environment of three different locations (two homes, two schools and two offices) in the city of Agra (Fig. 1). Sampling was carried out for three months for 24 h for personal exposure and ambient sampling. For personal monitoring the time duration contain the occupant׳s outdoor visits and their routine indoor interactions. The number of collected samples in a month were 12; six for ambient (i.e. two from homes, two from offices and two from schools) and six samples of personal exposure (i.e. two from homes, two from offices and two from schools) respectively. During the sampling duration parameters like CO_2_, temperature, relative humidity and air exchange rate were also measured on sampling site by using (YES- 205 and 206) Falcon indoor air quality monitor from (Young Environment Systems, Inc. 140-8771 Douglas St. Richmond, B.C. V6XV2 Canada). For chemical analysis twenty-four hour samples were collected of PM_2.5_ on 47 mm diameter, 2 µm pore size PTFE filter paper. Blank test background contamination was monitored by using operational blanks (unexposed filter papers), which were processed with field samples. The blank filters were taken thrice during every analysis. They were exposed in the field when the field-sampling box was opened to remove and replace field samples. Background contamination of heavy trace metals was determined by subtracting the field blank values from concentrations. Field blank values were very low, typically below or around the method detection limits. In this work, the background contamination was used to correct measurements. PM_2.5_ samples from ambient environment were collected with fine particulate medium volume dust sampler (APM 550, Envirotech), which runs at a constant flow rate of 16.6 L min^−1^ and for personal monitoring PEM (Personal Environmnetal Monitor, SKC Inc., USA) with Leland legacy sampling pump at 10 L/min, fitted within a waist pack to each individual participating. PEM uses 37-mm after-filter papers for the collection of PM_2.5_. Filters were handled only with tweezers coated with teflon tape to reduce the possibility of contamination. After collecting the loaded filters they were refrigerated, at about 4 °C, to limit the loss of semi volatile components. The total mass of filter paper was determined by weighing on a four-digit microbalance (Citizen, Model no. ISO 9001:2000) with precision of 2 mg and in the 220 mg range. Filters were conditioned for 48 h at a stable temperature (21±0.5 °C) and relative humidity (35±5%) before pre-sample and post-sample weighing. The collected filters were extracted by digesting in a 6–8 ml analytical grade (Merck) HNO_3_ (APHA, 1997) and kept on a hot plate at the temperature of 40–60 °C for 90 min. The solution was diluted up to 50 ml with distilled de-ionized water and stored in polypropylene sample bottles (these bottles were dipped in 2% HNO_3_) overnight before storing and then again dipped in deionized distilled water overnight to remove any impurities on these bottles. Analysis for eight trace metals Zinc (Zn), Lead (Pb), Nickel (Ni), Iron (Fe), Chromium (Cr), Cadmium (Cd), Manganese (Mn), and Copper (Cu) was done on Atomic Absorption Spectrophotometer (AAS) (Perkin Elmer, AAnalyst 100) regularly. In the present work survey samples were also collected from homes, schools and offices located in the urban environment of the Agra region. During the sampling 200 questionnaires were made to fill by the occupants (i.e. 100 from infants and 100 from adults), which also covered the sampling locations (i.e. two schools, two offices and two homes). The infants and adults who participated in questionnaire survey were the age group of (12–15 years and 30–50 years respectively). According to the responses by the respondents in our survey, the symptoms like irritation of the eyes, headaches, cold and flu, certain kind of allergies, sinus, mental fatigue, nausea and dizziness were reported (Fig. 2).

### 1.2. Particulate matter (PM_2.5_) and its chemical constituents

Table 1, lists the average concentrations of PM_2.5_ personal and ambient concentrations at home, school and office sites. During the sampling period the spatial settings revealed a rank order of higher concentrations (schools>homes>offices) with their means ranging from 112.28 to 140.24 µg m^−^^3^. On comparing the annual average PM_2.5_ concentration with National Ambient Air Quality Standard (40 μg m^−3^ annual average, respectively) [7] (Central Pollution Control Board), It was found to be 2.5–4 times higher. We also compared our values with World Health Organization [1] standards (10 µg m^−3^ annual mean respectively), our concentrations exceeded 11.5–14 times for PM_2.5_. Statistical summary of parameters like temperature, relative humidity, CO_2_ concentration and air exchange rate is shown in Table 2. The temperature ranged from 20.44 °C to 27.68 °C at all the sampling sites, whereas the relative humidity varied from 30.72% to 62.15%. The air exchange rate was recorded somewhat better at offices and homes in comparison to schools.

Characterization of PM_2.5_ components, including inorganic elements, is of central importance in proposing mechanisms for health effects and in source apportionment studies [2]. Values obtained by chemical analysis for the eight metals in PM_2.5_ particulate size collected from personal and ambient environments of home, school and office sites are also shown in Table 1 respectively. The sum of the eight parameters determined in PM_2.5_ personal and ambient environment at home sites ranged from 0.01–7.25 µg m^−^^3^ to 0.04–4.29 µg m^−3^ respectively. At school sites concentration ranged from 0.03–7.45 µg m^−3^ to 0.07–4.35 µg m^−3^, whereas at office sites, it ranged from 0.01–6.25 µg m^−3^ to 0.02–4.11 µg m^−3^ respectively. Above discussion shows a higher contribution of analyzed parameters in a personal and ambient environment of home and school sites in comparison to office sites.

### 1.3. Metal concentration and correlation analysis

Correlation analysis was performed to determine the relationship between personal and ambient sources among ionic species. Table 3 shows the interrelation between ionic species at the homes, schools and offices, during the sampling duration. This is important to note that the sample size used for the regression analysis in the present work was relatively small (*n*=36) and eight variables in each sample of PM_2.5_. Therefore it is possible that the correlation coefficient was significantly affected by one or two extremely high levels of values. For these reasons interpretation of the correlation values should be taken as suggestive rather than definitive. Fe presented the maximum concentration in indoor of at all the three sites followed by Cr, Pd, Zn, Ni, Cu, Cd, and Mn. The coefficient of variance (Cv) was in order of Fe>Cr>Pb>Zn>Ni>Cu>Cd>Mn. Similar kind of trends found in trace metal concentrations in all the three microenvironment, indicating one or more similar kind of sources contributing to these microenvironment, being present in similar kind of urban environment of city area. Correlation analysis was performed to determine the relationship between individual trace metals and to hypothesize probable sources on the assumption that two or more components may correlate either due to a common origin (Table 3). Zn and Ni showed good to strong correlation with Cr (*R*^2^=0.712 and 0.824) and Mn (*R*^2^=0.758 and 0.792) at home sites, (*R*^2^=0.876 and 0.996) and (*R*^2^=0.501 and 0.697) at school sites and (*R*^2^=0.798 and 0.975) and (*R*^2^=0.821 and 0.946) at office sites respectively. These correlations indicate smoking done by the occupants in the indoor working environment and incense burning could be the probable source of these trace metals ([3], Horemans and Grieken [4]). Fe also showed strong correlation with Cu at these three sites (*R*^2^=0.913, 0.921 and 0.902), followed by Cr with Mn (*R*^2^=0.736, 0.826 and 0.872) and Ni with Cr (*R*^2^=0.824, 0.501, and 0.821) respectively at these sites. Anthropogenic activities and use of different mechanical and electrical apparatus like computers, printers and photocopiers, etc. In the work environment can give rise emissions of such metals [5]. Ni also showed a correlation with Cd (*R*^2^=0.512, 0.501) at home and school sites which may be due to ambient sources.

### 1.4. Health effects

According to the responses by the respondents in our survey, the symptoms like irritation of the eyes, headaches, cold and flu, certain kind of allergies, sinus, mental fatigue, nausea and dizziness were reported. The values collected are shown in the form of percentage in Table 4. The most commonly reported symptoms were from the occupants of schools followed by homes and offices. In schools around 45–65%, in homes 35–53% and 20–52% people suffered from headaches, eyes and nose irritation, cold and flu and certain kind of allergy problems. Whereas, the problems like sinus, mental fatigue, nausea and dizziness were normal. As the schools houses and offices are located near the busy roads (Fig. 1), vehicular emission causes the formation of fine particulate that penetrates indoors (higher values of *r*) and in turn is supposed to increase health related problems [2]. In indoors, sources like smoking, incense burning, various indoor human activities are also responsible for the cause of various health effects in occupants. Further, their inability to improve their environment is also a source of stress, which can contribute to the enhancement of their symptoms [6]. Thus, there is an immediate need to address the issue of particles, especially fine particles and their related toxicity in different indoor microenvironments.

## Figures and Tables

**Table 1:** Average concentrations (µg m^−3^) of analysed parameters in PM_2.5_ personal and ambient environment of homes, schools and offices from December 2013 to February 2014.

Homes	PM_2.5_ personal							PM_2.5_ ambient environment					
	Mean		SD		Max		Min	Mean		SD		Max	Min
PM conc.	135.28		44.86		180.27		105.90	123.79		35.32		184.67	81.43
Fe	2.61		2.07		7.25		0.71	1.46		1.60		4.29	0.12
Pb	1.03		1.17		3.73		0.03	1.20		0.66		2.24	0.19
Mn	0.06		0.08		0.29		0.01	0.28		0.32		0.54	0.15
Cu	0.42		0.37		1.14		0.04	0.41		0.49		1.18	0.03
Ni	0.62		0.60		1.93		0.03	0.73		0.98		3.05	0.04
Cr	2.45		1.50		6.21		0.31	0.64		1.05		3.13	0.04
Zn	0.55		0.52		2.01		0.02	1.85		2.33		3.58	0.27
Cd	0.38		0.13		0.43		0.22	0.40		0.14		0.48	0.27
		
Schools													
PM conc.	140.24	42.21		211.24		122.35		128.45	37.58		192.24		76.24
Fe	2.78	2.11		7.45		0.82		1.57	1.68		4.35		0.16
Pb	1.16	1.28		3.89		0.12		1.45	0.85		2.56		0.24
Mn	0.10	0.09		0.35		0.03		0.26	0.38		0.56		0.16
Cu	0.48	0.35		1.06		0.05		0.42	0.47		1.20		0.06
Ni	0.72	0.63		2.04		0.05		0.75	0.87		3.32		0.09
Cr	3.01	1.75		6.35		0.38		0.75	1.07		3.22		0.07
Zn	0.59	0.57		2.00		0.03		1.88	2.32		3.68		0.24
Cd	0.37	0.11		0.42		0.21		0.45	0.16		0.47		0.25
		
Offices													
PM conc.	125.78	40.24		162.58		95.78		112.28	36.45		185.77		80.44
Fe	2.45	1.78		6.25		0.53		1.38	1.57		4.11		0.11
Pb	1.02	1.12		3.59		0.03		1.15	0.59		2.13		0.15
Mn	0.05	0.07		0.25		0.01		0.22	0.31		0.53		0.11
Cu	0.38	0.35		1.09		0.02		0.37	0.45		1.11		0.03
Ni	0.61	0.57		1.78		0.01		0.67	0.88		2.98		0.03
Cr	2.42	1.45		6.00		0.27		0.58	0.98		3.03		0.02
Zn	0.45	0.47		1.85		0.01		1.78	2.12		3.50		0.21
Cd	0.32	0.11		0.35		0.20		0.35	0.09		0.36		0.21


**Table 2:** Statistical summary of, temperature, CO_2_, humidity and air exchange rate during the sampling duration at homes, schools and offices.

Schools	CO_2_ PPM	Temp. (°C)	Rel. humidity (%)	Air exchange rate (h^−1^)	Homes	CO_2_ PPM	Temp. (°C)	Rel. humidity (%)	Air exchange rate (h^−1^)	Offices	CO2 PPM	Temp. (°C)	Rel. humidity (%)	Air exchange rate (h^−1^)
Average	545.97	24.59	49.31	2.45		423.53	24.58	58.76	4.57		373.37	23.95	32.02	5.24
SD	10.78	2.93	1.03	0.37		7.41	3.68	4.39	0.27		19.18	3.18	3.59	0.10
Maximum	555.49	26.83	50.39	2.86		432.78	27.68	62.15	4.86		389.45	26.67	35.83	5.36
Minimum	534.27	21.27	48.34	2.14		416.35	20.50	55.37	4.32		352.15	20.44	30.72	5.17
Geo. mean	539.48	24.47	48.75	2.35		417.07	24.38	57.15	4.22		368.45	23.38	31.02	5.11

**Table 3:** Correlation matrix at home, school and office sites.

Homes								
Metals	Zn	Pd	Ni	Fe	Cr	Cd	Mn	Cu
Zn	1.000							
Pd	0.455	1.000						
Ni	0.687⁎	0.264	1.000					
Fe	0.211	0.421	0.475	1.000				
Cr	0.712⁎	0.487	0.824⁎	0.664⁎	1.000			
Cd	0.216	0.124	0.512⁎	0.227	0.427	1.000		
Mn	0.758⁎	0.348	0.792⁎	0.216	0.736⁎	0.178	1.000	
Cu	0.372	0.367	0.218	0.913⁎⁎	0.689⁎	0.246	0.215	1.000
Schools								
Zn	1.000							
Pd	0.368	1.000						
Ni	0.497	0.168	1.000					
Fe	0.318	0.336	0.587⁎	1.000				
Cr	0.876⁎	0.421	0.501⁎	0.478	1.000			
Cd	0.325	0.248	0.501⁎	0.246	0.416	1.000		
Mn	0.996⁎⁎	0.485	0.226	0.364	0.826⁎	0.325	1.000	
Cu	0.116	0.167	0.697⁎	0.921⁎⁎	0.348	0.348	0.139	1.000
Offices								
Zn	1.000							
Pd	0.358	1.000						
Ni	0.826⁎	0.276	1.000					
Fe	0.296	0.331	0.379	1.000				
Cr	0.798⁎	0.167	0.821⁎	0.334	1.000			
Cd	0.058	0.256	0.428	0.387	0.342	1.000		
Mn	0.975⁎⁎	0.428	0.946⁎⁎	0.421	0.872⁎	0.315	1.000	
Cu	0.018	0.348	0.167	0.902⁎	0.246	0.216	0.224	1.000

⁎ *P*=0.05.

⁎⁎ *P*=0.01.

**Table 4:** Relationship of health effects and particulate levels.

Questionnaire survey analysis in (%). *Short-term health effects*	Homes	Schools	Offices
1. Headaches	53	65	52
2. Eyes and nose irritation	53	60	48
3. Cold and flu	50	62	38
4. Allergies	35	45	20
5. Sinus	20	22	14
6. Mental fatigue	28	35	22
7. Nausea and dizziness	18	20	13
