# Supplementary material for: Personal and ambient PM2.5 exposure assessment in the city of Agra
Source: Data Brief. 2016 Jan 6;6:495–502. doi: 10.1016/j.dib.2015.12.040 (PMC4716460; doi:10.1016/j.dib.2015.12.040)
Supplement: Supplementary file 1 — Supplementary material [file mmc1.zip › Table 1.doc]

Table 1. Average concentrations (ugm-3) of analysed parameters in PM_2.5_ personal and ambient environment of homes, schools and offices from December 2013 to February 2014.

| Homes | PM_2.5_ Personal | | | | | | | | PM_2.5_ Ambient Environment | | | | | |
| --- | --- | --- | --- | --- | --- | --- | --- | --- | --- | --- | --- | --- | --- | --- |
|  | Mean | | SD | | Max | | Min | | Mean | | SD | | Max | Min |
| PM Conc. | 135.28 | | 44.86 | | 180.27 | | 105.90 | | 123.79 | | 35.32 | | 184.67 | 81.43 |
| Fe | 2.61 | | 2.07 | | 7.25 | | 0.71 | | 1.46 | | 1.60 | | 4.29 | 0.12 |
| Pb | 1.03 | | 1.17 | | 3.73 | | 0.03 | | 1.20 | | 0.66 | | 2.24 | 0.19 |
| Mn | 0.06 | | 0.08 | | 0.29 | | 0.01 | | 0.28 | | 0.32 | | 0.54 | 0.15 |
| Cu | 0.42 | | 0.37 | | 1.14 | | 0.04 | | 0.41 | | 0.49 | | 1.18 | 0.03 |
| Ni | 0.62 | | 0.60 | | 1.93 | | 0.03 | | 0.73 | | 0.98 | | 3.05 | 0.04 |
| Cr | 2.45 | | 1.50 | | 6.21 | | 0.31 | | 0.64 | | 1.05 | | 3.13 | 0.04 |
| Zn | 0.55 | | 0.52 | | 2.01 | | 0.02 | | 1.85 | | 2.33 | | 3.58 | 0.27 |
| Cd | 0.38 | | 0.13 | | 0.43 | | 0.22 | | 0.40 | | 0.14 | | 0.48 | 0.27 |
| Schools | PM_2.5_ Personal | | | | | | | PM_2.5_ Ambient Environment | | | | | | |
|  | Mean | SD | | Max | | Min | | Mean | | SD | | Max | | Min |
| PM Conc. | 140.24 | 42.21 | | 211.24 | | 122.35 | | 128.45 | | 37.58 | | 192.24 | | 76.24 |
| Fe | 2.78 | 2.11 | | 7.45 | | 0.82 | | 1.57 | | 1.68 | | 4.35 | | 0.16 |
| Pb | 1.16 | 1.28 | | 3.89 | | 0.12 | | 1.45 | | 0.85 | | 2.56 | | 0.24 |
| Mn | 0.10 | 0.09 | | 0.35 | | 0.03 | | 0.26 | | 0.38 | | 0.56 | | 0.16 |
| Cu | 0.48 | 0.35 | | 1.06 | | 0.05 | | 0.42 | | 0.47 | | 1.20 | | 006 |
| Ni | 0.72 | 0.63 | | 2.04 | | 0.05 | | 0.75 | | 0.87 | | 3.32 | | 0.09 |
| Cr | 3.01 | 1.75 | | 6.35 | | 0.38 | | 0.75 | | 1.07 | | 3.22 | | 0.07 |
| Zn | 0.59 | 0.57 | | 2.00 | | 0.03 | | 1.88 | | 2.32 | | 3.68 | | 0.24 |
| Cd | 0.37 | 0.11 | | 0.42 | | 0.21 | | 0.45 | | 0.16 | | 0.47 | | 0.25 |
| Offices | PM_2.5_ Personal | | | | | | | PM_2.5_ Ambient Environment | | | | | | |
|  | Mean | SD | | Max | | Min | | Mean | | SD | | Max | | Min |
| PM Conc. | 125.78 | 40.24 | | 162.58 | | 95.78 | | 112.28 | | 36.45 | | 185.77 | | 80.44 |
| Fe | 2.45 | 1.78 | | 6.25 | | 0.53 | | 1.38 | | 1.57 | | 4.11 | | 0.11 |
| Pb | 1.02 | 1.12 | | 3.59 | | 0.03 | | 1.15 | | 0.59 | | 2.13 | | 0.15 |
| Mn | 0.05 | 0.07 | | 0.25 | | 0.01 | | 0.22 | | 0.31 | | 0.53 | | 0.11 |
| Cu | 0.38 | 0.35 | | 1.09 | | 0.02 | | 0.37 | | 0.45 | | 1.11 | | 0.03 |
| Ni | 0.61 | 0.57 | | 1.78 | | 0.01 | | 0.67 | | 0.88 | | 2.98 | | 0.03 |
| Cr | 2.42 | 1.45 | | 6.00 | | 0.27 | | 0.58 | | 0.98 | | 3.03 | | 0.02 |
| Zn | 0.45 | 0.47 | | 1.85 | | 0.01 | | 1.78 | | 2.12 | | 3.50 | | 0.21 |
| Cd | 0.32 | 0.11 | | 0.35 | | 0.20 | | 0.35 | | 0.09 | | 0.36 | | 0.21 |
