# Supplementary material for: Personal and ambient PM2.5 exposure assessment in the city of Agra
Source: Data Brief. 2016 Jan 6;6:495–502. doi: 10.1016/j.dib.2015.12.040 (PMC4716460; doi:10.1016/j.dib.2015.12.040)
Supplement: Supplementary file 1 — Supplementary material [file mmc1.zip › Table 2.doc]

Table 2. Statistical summary of, temperature, CO_2_, humidity and air exchange rate during the sampling duration at homes, schools and offices.

| Schools | CO_2_  PPM | Temp.  °C | Rel.  Humidity  % | Air exchange  rate h^-1^ | Homes | CO_2_  PPM | Temp.  °C | Rel.  Humidity  % | Air exchange  rate h-1 | Offices | CO2  PPM | Temp.  °C | Rel.  Humidity  % | Air exchange  rate h-1 |
| --- | --- | --- | --- | --- | --- | --- | --- | --- | --- | --- | --- | --- | --- | --- |
| Average | 545.97 | 24.59 | 49.31 | 2.45 |  | 423.53 | 24.58 | 58.76 | 4.57 |  | 373.37 | 23.95 | 32.02 | 5.24 |
| Sd | 10.78 | 2.93 | 1.03 | 0.37 |  | 7.41 | 3.68 | 4.39 | 0.27 |  | 19.18 | 3.18 | 3.59 | 0.10 |
| Maximum | 555.49 | 26.83 | 50.39 | 2.86 |  | 432.78 | 27.68 | 62.15 | 4.86 |  | 389.45 | 26.67 | 35.83 | 5.36 |
| Minimum | 534.27 | 21.27 | 48.34 | 2.14 |  | 416.35 | 20.50 | 55.37 | 4.32 |  | 352.15 | 20.44 | 30.72 | 5.17 |
| Geo.Mean | 539.48 | 24.47 | 48.75 | 2.35 |  | 417.07 | 24.38 | 57.15 | 4.22 |  | 368.45 | 23.38 | 31.02 | 5.11 |
