# Supplementary material for: Personal and ambient PM2.5 exposure assessment in the city of Agra
Source: Data Brief. 2016 Jan 6;6:495–502. doi: 10.1016/j.dib.2015.12.040 (PMC4716460; doi:10.1016/j.dib.2015.12.040)
Supplement: Supplementary file 1 — Supplementary material [file mmc1.zip › Table 3.doc]

Table 3. Correlation matrix at home, school and office sites.

| Homes |  |  |  |  |  |  |  |  |
| --- | --- | --- | --- | --- | --- | --- | --- | --- |
| Metals | Zn | Pd | Ni | Fe | Cr | Cd | Mn | Cu |
| Zn | 1.000 |  |  |  |  |  |  |  |
| Pd | 0.455 | 1.000 |  |  |  |  |  |  |
| Ni | 0.687* | 0.264 | 1.000 |  |  |  |  |  |
| Fe | 0.211 | 0.421 | 0.475 | 1.000 |  |  |  |  |
| Cr | 0.712* | 0.487 | 0.824* | 0.664* | 1.000 |  |  |  |
| Cd | 0.216 | 0.124 | 0.512* | 0.227 | 0.427 | 1.000 |  |  |
| Mn | 0.758* | 0.348 | 0.792* | 0.216 | 0.736* | 0.178 | 1.000 |  |
| Cu | 0.372 | 0.367 | 0.218 | 0.913** | 0.689* | 0.246 | 0.215 | 1.000 |
| Schools |  |  |  |  |  |  |  |  |
| Zn | 1.000 |  |  |  |  |  |  |  |
| Pd | 0.368 | 1.000 |  |  |  |  |  |  |
| Ni | 0.497 | 0.168 | 1.000 |  |  |  |  |  |
| Fe | 0.318 | 0.336 | 0.587* | 1.000 |  |  |  |  |
| Cr | 0.876* | 0.421 | 0.501* | 0.478 | 1.000 |  |  |  |
| Cd | 0.325 | 0.248 | 0.501* | 0.246 | 0.416 | 1.000 |  |  |
| Mn | 0.996** | 0.485 | 0.226 | 0.364 | 0.826* | 0.325 | 1.000 |  |
| Cu | 0.116 | 0.167 | 0.697* | 0.921** | 0.348 | 0.348 | 0.139 | 1.000 |
| Offices | | |  |  |  |  |  |  |
| Zn | 1.000 |  |  |  |  |  |  |  |
| Pd | 0.358 | 1.000 |  |  |  |  |  |  |
| Ni | 0.826* | 0.276 | 1.000 |  |  |  |  |  |
| Fe | 0.296 | 0.331 | 0.379 | 1.000 |  |  |  |  |
| Cr | 0.798* | 0.167 | 0.821* | 0.334 | 1.000 |  |  |  |
| Cd | 0.058 | 0.256 | 0.428 | 0.387 | 0.342 | 1.000 |  |  |
| Mn | 0.975** | 0.428 | 0.946** | 0.421 | 0.872* | 0.315 | 1.000 |  |
| Cu | 0.018 | 0.348 | 0.167 | 0.902* | 0.246 | 0.216 | 0.224 | 1.000 |

*p = 0.05, **p = 0.01.
