# Supplementary material for: Personal and ambient PM2.5 exposure assessment in the city of Agra
Source: Data Brief. 2016 Jan 6;6:495–502. doi: 10.1016/j.dib.2015.12.040 (PMC4716460; doi:10.1016/j.dib.2015.12.040)
Supplement: Supplementary file 1 — Supplementary material [file mmc1.zip › Table 4.doc]

Table 4. Relationship of health effects and particulate levels.

| Questionnaire survey analysis in (percentage).  *Short-term health effects* | | Homes | Schools | Offices |
| --- | --- | --- | --- | --- |
| 1. Headaches |  | 53 | 65 | 52 |
| 2. Eyes and nose irritation |  | 53 | 60 | 48 |
| 3. Cold and flu |  | 50 | 62 | 38 |
| 4. Allergies |  | 35 | 45 | 20 |
| 5. Sinus |  | 20 | 22 | 14 |
| 6. Mental fatigue |  | 28 | 35 | 22 |
| 7. Nausea and dizziness |  | 18 | 20 | 13 |
