# Supplementary material for: Personal and ambient PM2.5 exposure assessment in the city of Agra
Source: Data Brief. 2016 Jan 6;6:495–502. doi: 10.1016/j.dib.2015.12.040 (PMC4716460; doi:10.1016/j.dib.2015.12.040)
Supplement: Supplementary file 1 — Supplementary material [file mmc1.zip › Figure 1.doc]

Figure1. Map of Agra showing sampling sites.
